# Effects of Chronic Hypoxic Environment on Cognitive Function and Neuroimaging Measures in a High-Altitude Population

**DOI:** 10.3389/fnagi.2022.788322

**Published:** 2022-05-06

**Authors:** Ya Qing Zhang, Wen juan Zhang, Jin hao Liu, Wei zhong Ji

**Affiliations:** Qinghai Provincial People’s Hospital, Xining, China

**Keywords:** altitude, hypoxia exposure, cognitive function, neuropsychological assessment, magnetic resonance imaging

## Abstract

**Objective:**

This study aimed to investigate the effects of long-term hypoxic environment exposure on cognitive ability and neuroimaging characteristics in a highland population in China.

**Methods:**

Health system workers in Maduo County (4,300 m above sea level) and Minhe County (1,700 m above sea level) were selected as research participants and divided into a high-altitude (HA) group and low-altitude (LA) group, respectively. Cognitive ability was assessed using the Montreal Cognitive Assessment (MoCA), Verbal Fluency Test (VFT), Symbol Digit Modalities Test (SDMT), Trail Making Test A and B (TMT), Digit Span Test (DST), and Rey Auditory Verbal Learning Test (RAVLT). All participants underwent a magnetic resonance imaging (MRI) scan, resting state functional MRI scan, and diffusion tensor imaging to clarify changes in regional gray matter (GM) volume, anisotropy index (FA), local consistency (ReHo), and low-frequency oscillation amplitude (ALFF).

**Results:**

The HA group had significantly lower MoCA, DST, VFT, RAVLT, and TMT scores compared to the control group. No significant differences were found in SDMT score. Furthermore, compared to the LA group, the HA group had significantly lower GM density of the left olfactory cortex, right medial orbital superior frontal gyrus, bilateral insula, left globus pallidus, and temporal lobe (left superior temporal gyrus temporal pole, bilateral middle temporal gyrus temporal pole, and right middle temporal gyrus). In terms of FA, compared with the LA group, the HA group had lower values for the corpus callosum, corpus callosum knee, bilateral radiative corona, and left internal capsule. The HA group had lower ALFF values of the left cerebellum, left putamen, left orbital inferior frontal gyrus, and left precuneus, but higher ALFF values of the left fusiform gyrus, bilateral inferior temporal gyrus, left orbital superior frontal gyrus and medial superior frontal gyrus, compared to the LA group. There was no significant group difference in ReHo values.

**Conclusion:**

Our findings suggest that a chronic hypoxic environment can induce extensive cognitive impairment. Decreased GM density in multiple brain regions, damaged nerve fibers, and unbalanced neuronal activity intensity in different brain regions may be the structural and functional basis of cognitive impairment due to hypoxia.

## Introduction

With the enhancement of social, economic, and military activities in the plateau region of China in recent years, studies on the effects of a high altitude and low oxygen environment on human brain function have gradually increased. While many studies focus on the effects of acute altitude exposure on the human brain, there are relatively few studies on the effects of chronic hypoxia on human brain function. Most prior studies used a single cognitive test scale, and are thus unable to assess specific effects on memory, learning, spatial perception, execution, behavior, etc., and did not account for potential effects of nationality, education level, economic status, mental state, and other factors. Furthermore, the effects of long-term hypoxic environmental exposure on brain structure and function specifically in the plateau population are unknown.

At present, it is believed that the influence of a high altitude and low oxygen environment on cognitive function is reflected by changes in brain structure and metabolism to some extent. In recent years, functional magnetic resonance imaging (fMRI) has been widely used in cognitive neuroscience research (Zaca et al., 2014). A comparative MRI study found that the gray matter (GM) density of the bilateral prefrontal cortex and bilateral insula cortex was significantly reduced in people living at high altitudes (Di Paola et al., 2008). Zatorre et al. (2012) found that the decrease in GM volume may be related to the anaerobic metabolism by-products of brain tissues during of chronic hypoxia. A study of high-altitude migrants found that the intensity of speech working memory-related responses in the inferior frontal gyrus, middle frontal gyrus, and cerebellum was significantly lower than that of sea level migrants (Yan et al., 2011a). Taken together, these findings suggest that chronic hypoxia may lead to functional changes in brain regions related to working memory. Chen et al. (2016) used fMRI to compare changes in resting brain function in HA migrants. Local consistency (ReHo) analysis showed that ReHo significantly increased in the right lower sensorimotor cortex in HA migrants, and that the change in value was correlated with response time during a memory search task. In addition, bilateral visual cortex signals were significantly enhanced and correlated with subjects’ hemoglobin concentration, suggesting that chronic hypoxia exposure may affect the synchronization and connectivity of spontaneous neural activity, which may be the functional brain basis of cognitive changes. In a fMRI study, Chen et al. (2017) found that chronic hypoxic exposure may cause pathological changes in neurons in the inner capsule and other areas and decrease GM volume in the left putamen, suggesting that a chronic hypoxic environment may cause irreversible damage to neurons and thus cause changes in brain structure. MRI was used to detect brain injury in athletes climbing Mount Qomolangma (also known as Mount Everest), and magnetic resonance spectroscopy was used to measure the metabolic rate of brain tissue. Most athletes showed cortical atrophy, but no changes in the metabolic rate were found.

As most previous studies used only one neuroimaging method to study cognitive function in populations with chronic hypoxic exposure, although some applied multimodal magnetic resonance technology, there are issues such as inconsistent conclusions and inconsistent evaluation criteria. Furthermore, there are no studies of people living at a high altitude that combined multiple psychological cognitive measures with multimodal magnetic resonance technology. Therefore, based on conclusions from our previous animal experiments on cognitive impairment in chronic hypoxia (Ji et al., 2021a,b), we propose the following research hypothesis: indigenous groups living in plateau regions may show different degrees of cognitive impairment, which may coexist with brain structure GM abnormalities and functional abnormalities of the brain. In order to test this hypothesis, we adopted a cluster sampling method to assess various cognitive abilities in individuals living at a HA region (4,300 m) and low-altitude region (1,600 m) in Qinghai Province, China. We further conducted multimodal MRI for all participants to determine the imaging characteristics of cognitive impairment caused by chronic hypoxia exposure.

## Materials and Methods

### Study Design

Medical staff in Maduo County People’s Hospital of Goluo Prefecture in Qinghai Province were selected by the cluster sampling method as the high-altitude group (HA group, 4,300 m), resulting in a total of 49 participants. Medical staff from Haidong Citizens and the Second People’s Hospital of Qinghai Province were selected as the low-altitude group (LA group, 1,600 m), resulting in a total of 43 participants. Thus, the total sample size for this study is 92 people.

First, participants underwent complete neuropsychological testing in a quiet environment, and a diagnosis was made by the deputy chief physician of neurology department of Qinghai Provincial People’s Hospital. Participants were further examined by multimodal MRI, and a diagnosis was made by an associate chief physician specializing in imaging. This study was approved by the Ethics Committee of Qinghai Provincial People’s Hospital and all participants provided written informed consent.

### Study Participants

The inclusion criteria were as follows: (1) 20–50 years old; (2) a high school education or above; (3) local residence (living in the region for more than three generations); (4) barrier-free Chinese language comprehension and writing; (5) having unrestricted work and social activities; and (6) signing informed consent and agreeing to complete cognitive function testing and brain MRI examination. The exclusion criteria were: (1) cognitive decline due to cerebrovascular or nervous system diseases (including vascular dementia); (2) cognitive decline caused by poisoning or systemic diseases; (3) cognitive decline caused by mental diseases such as anxiety and depression; (4) cognitive decline caused by a recent cold, acute disease, chronic inflammation, metabolic disease, infectious disease, etc.; (5) chronic diseases and familial genetic diseases; and (6) cognitive decline due to other reasons.

### General Data Collection

We collected general information about the participants, including age, sex, ethnicity, education level, smoking history, alcohol consumption history, etc. The criterion for a history of smoking was an average of more than five cigarettes per day. The criterion for a history of alcohol consumption was an average of more than 50 ml alcohol/day.

### Cognitive Function Assessment

We used the Montreal Cognitive Assessment Scale (MoCA), Digit Span Test (DST), Symbol Digit Modalities Test (SDMT), Trail Making Test A and B (TMT), Verbal Fluency Test (VFT), and Rey Auditory Verbal Learning Test (RAVLT) to comprehensively assess cognitive function in the study participants. The HA and LA groups were tested in June and July 2019, respectively. All tests were administered by professionally trained associate chief physicians of the department of neurology in a quiet environment.

Statistical analysis: SPSS20.0 statistical software was used for data analysis. In continuous variable data, data subject to normal distribution or approximately normal distribution was represented by mean ± standard deviation (*x* ± SD), Comparison between the two groups was performed by *t*-test. Qualitative data was represented by percentage (%). Non-parametric tests (chi-square test) was used for continuous variable data that did not obey normal distribution, and *p* < 0.05 was considered statistically significant.

### Neuroimaging Assessment

According to the neuropsychological assessment, 34 people (69.39%; 14 men and 20 women) in the HA group met the diagnostic criteria of mild cognitive impairment (MCI; Winblad et al., 2004). The remaining 15 participants (6 men and 9 women) in this group had normal cognitive function. Sixteen people (37.21%; 7 men and 9 women) in the LA group met the diagnostic criteria of MCI. The remaining 27 participants (12 men and 15 women) in this group had normal cognitive function.

Further MRI neuroimaging assessment was arranged and relevant data were collected. This included structural MRI [3DT1, diffusion tensor imaging (DTI)] and resting-state functional MRI. A Siemens SLYRA 3.0T NMR instrument was used to collect data with 20-channel head coil. All participants were assessed at the Imaging Center of Qinghai Provincial People’s Hospital and instructed to stay awake and keep their heads as still as possible.

#### Structural Magnetic Resonance Imaging

Structural MRI mainly used standard T1-weighted 3D imaging and DTI data to analyze structural brain changes between the two groups. 3D T1 main parameters: echo time was set as 2.43 ms; Repeat time 1,900 ms; Turning Angle 9°C;Field of view of 256 mm; Matrix 256; Section thickness 1 mm; Section clearance 0.5 mm; Section number 176; Voxel size: 1.0 mm × 1.0 mm × 1.0 mm; The main parameters of DTI: the diffusion weighted gradient is applied in 30 non-parallel directions (B value: 0 ∼ 1,000); Repetition time 9,500 ms; Echo time 92 ms; The field of view is set to 220 mm; Layer thickness 4 mm; Layer spacing 1.2 mm; Matrix 128.

##### 3D T1-Weighted Image Processing and Analysis

Using Matlab SPM12 (R2019b)^1^ calculation of anatomical toolkit (CAT12)^2^ for T1 weighted image preprocessing. Statistical analysis was performed using R language.

Data pretreatment: The T1 image space was registered into tissue probability maps, which was divided into three parts, GM, white matter (WM), and cerebrospinal fluid. The GM image was smoothed by 8 mm Gaussian kernel function. Image quality was assessed by visual inspection and the ratio of weighted average image quality index to average correlation coefficient.

Statistical analysis: The cortex was divided into 116 different brain regions using the Automatic Labeling (AAL) template. After the GM density of each brain region was obtained, the total intracranial volume was calculated and used as a control variable. The GM density of the HA group and LA group was tested using the two-sample *t*-test, and the regions with significant differences in GM density were obtained. Using the voxel-based morphometry (VBM) method, the changes of GM density and WM density of each voxel in the MRI were quantitatively measured.

##### Diffusion Tensor Imaging Image Processing and Analysis

Tract-based spatial statistics (TBSS) was used to analyze the DTI image data. Using MRIcron, we converted the DICOM file to the NIfTI format. According to the FSL instructions^3^ for DTI data preprocessing, feature extraction, fractional anisotropy (FA), skeleton generation, and statistical analysis were performed.

Data pretreatment: extraction of b0, scalp stripping, eddy current correction, and tensor fitting.

Feature extraction: FA is an indicator to quantify the directional strength of local regional structures.

Skeleton generation: Non-linear registration of individual FA to template FMRIB58_FA; The FA of all the research objects was averaged and the fiber bundle skeleton template was generated. The skeleton diagram of individual FA fiber bundles was calculated.

Statistical analysis: The two-sample *t*-test (with threshold-free cluster enhancement) was used to compare the FA values of the voxels of the fiber bundle skeleton in the HA group and the LA group, and the regions showing significant inter-group differences were obtained.

#### rs-fMRI

rs-fMRI was used to evaluate brain functional network changes. This study used the local consistency (ReHo), low frequency oscillation amplitude (ALFF), which measured the given voxel with time series similarity between the neighboring voxel, to explore the effect of chronic hypoxic exposure on local connectivity, spontaneous brain activity. After data collection, post-processing was performed.

DPARSF under DPABI^4^ based on Matlab (R2019b) was used to preprocess and extract features (ALFF, ReHo) from functional data, and SPM12 was used for statistical analysis.

Data preprocessing: 5 time points before the functional image scanning were removed, followed by time correction, head movement correction, and denoising, etc. Finally, DARTEL method was used for spatial standardization.

Feature extraction: after space standardization, bandpass filtering (0.01–0.1 Hz), and then calculating the low-frequency amplitude (ALFF, the energy of low-frequency signal reflects the strength of neuronal activity) and smoothing; Local consistency (ReHo, reflecting the consistency of neural activity) was calculated after spatial normalization and using 4 mm Gaussian kernel smoothing.

Statistical analysis: Using the two-sample *t*-test (*p* < 0.001, the cluster size > 50) The differences of ALFF and ReHo values between the high altitude group and the low altitude group were obtained.

## Results

### Comparison of General Information

A total of 92 subjects meeting the inclusion criteria were included in this study (49 in the HA group and 43 in the LA group). As shown in Table 1, there were no significant differences between the two groups in terms of age, sex, education, smoking, and drinking (all *p* > 0.05). However, there was a significant difference in ethnic composition between the two groups (*p* < 0.05).

**TABLE 1:** General characteristics of the two groups.

Characteristics	HA	LA	*t*/*χ^2^*	*P*
Age/years (*x* ± *s*)	30.88 ± 5.98	32.88 ± 6.77	−1.510	0.135
Gender (Male)	20 (49)	19 (43)	0.106	0.744
Education/years	14.75 ± 1.98	15.13 ± 1.72	−0.985	0.327
**Ethnic composition**				
Han	13 (26.53)	24 (55.81)	19.878	0.000
Tibetan	29 (59.18)	6 (13.95)		
Other	7 (14.29)	13 (30.23)		
Smoking	14 (28.57)	15 (34.88)	0.423	0.516
Alcohol	11 (22.45)	14 (32.56)	1.183	0.277

### Comparison of Cognitive Function

Neuropsychological scale scores for the two groups are shown in Table 2. Compared with the LA group, the comprehensive cognitive function score of the HA group was significantly lower (*p* < 0.05). Executive function and attention were decreased in the HA group (*p* < 0.05). In addition, total cognitive function and executive function, attention, memory function, and visuospatial function were decreased in the HA group (all *p* < 0.05). There was no difference in average age between the cognitively impaired and non-cognitively impaired persons.

**TABLE 2:** Neuropsychological scale scores for subjects in the two groups.

Scale	HA	LA	*t*/*χ^2^*	*p*
MoCA	25.29 ± 2.25	26.49 ± 1.97	−2.707	0.008
DST	10.35 ± 1.49	11.21 ± 1.41	−2.839	0.006
SDMT	45.45 ± 4.84	44.26 ± 4.43	1.227	0.223
TMT(A)	56.29 ± 8.02	52.93 ± 6.28	2.213	0.029
TMT(B)	120.63 ± 14.87	114.40 ± 12.77	2.143	0.035
VFT	26.55 ± 4.28	28.51 ± 4.18	−2.215	0.029
RAVLT	40.90 ± 5.32	43.37 ± 4.46	−2.400	0.018
The incidence of MCI (MoCA)	34/49 (69.39)	16/43 (37.21)	9.558	0.002

It is worth noting that there are ethnic differences between the HA group and the LA group, as there are more Tibetans in the HA group. However, after reviewing the literature, we found that the prevalence of Alzheimer’s disease (AD) in the elderly Tibetan population in Qinghai Province was significantly lower than that at the national level, However, in our study, we found that the incidence of MCI was higher in the HA and LA group (all people <50 years old) than in the LA group. This result suggests that the higher incidence of MCI in the HA group was associated with long-term chronic hypoxic exposure, not with ethnicity. It also suggests that there may be different pathophysiological mechanisms between AD and MCI induced by chronic hypoxia.

### Comparison of Structural Magnetic Resonance Imaging

Structural imaging can reflect changes in the GM and WM volume of brain tissue. Based on the 3D T1-weighted imaging and DTI results, the GM density and FA of WM of people living in high altitudes show significant changes, indicating that chronic hypoxia has a significant impact on brain structure.

#### Gray Matter Density Changes

**FIGURE 1:**
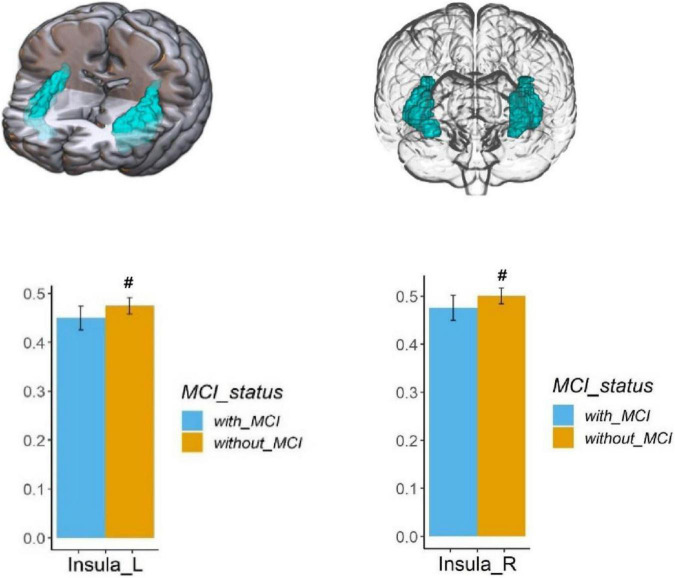
Regional GM density comparison between the HA (with MCI) and LA (without MCI) groups. Above: The blue area shows the reduced GM density of the bilateral insula (*P* < 0.05, Bonferroni corrected). Below: The bars represent statistical comparison of the bilateral insular GM density between the HA group and LA group. L – left hemisphere, R – right hemisphere.

**FIGURE 2:**
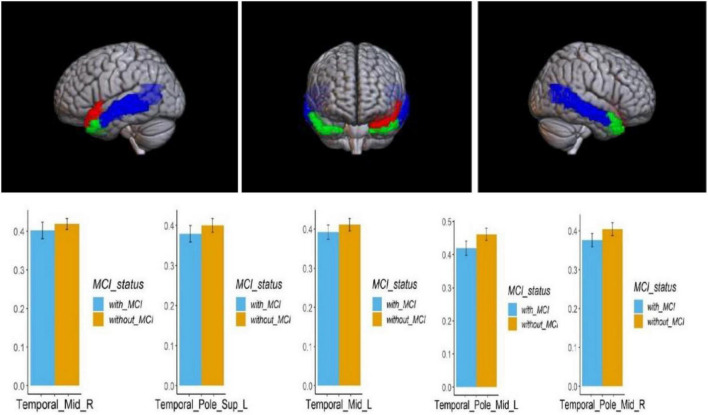
Regional comparison of the GM density between the HA (with MCI) and LA (without MCI) groups. The red area above represents the temporal pole of the left superior temporal gyrus; The blue area represents the bilateral middle temporal gyrus; The green area represents the bilateral middle temporal gyrus and temporal pole (*P* < 0.05, Bonferroni corrected). The bars represent the comparison of GM density in the temporal pole of the left superior temporal gyrus, bilateral superior temporal gyrus, and bilateral middle temporal gyrus between the HA group and LA group. L – left hemisphere, R – right hemisphere.

**FIGURE 3:**
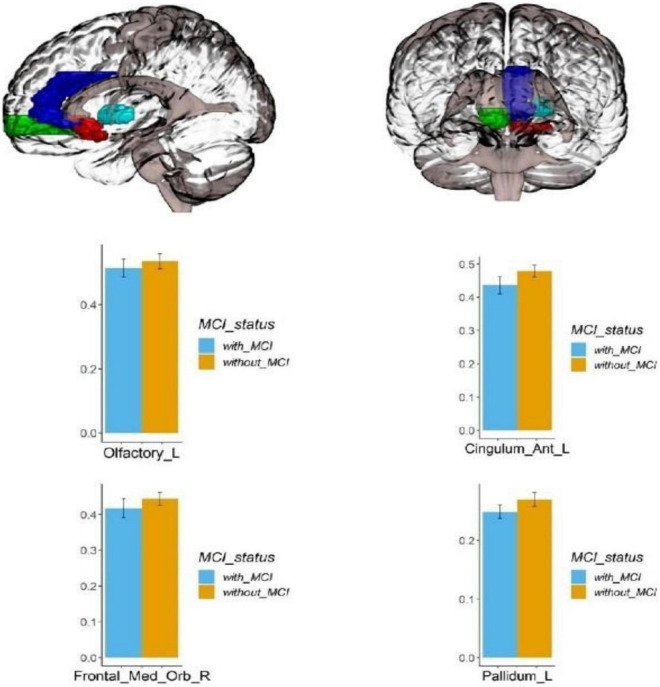
Regional GM density comparison between the HA (with MCI) and LA (without MCI) groups. **Left**: The red area represents the left occipital lobe; The dark blue area represents the left cingulate gyrus; The green area represents the right orbital superior frontal gyrus; The light blue area represents the left globus pallidus; (*p* < 0.05, Bonferroni corrected). Below **right**: bars represent statistical comparison of gray matter density in the left olfactory cortex, left cingulate gyrus, right orbital superior frontal gyrus and left globus pallidus between the HA and LA groups. L – left hemisphere, R – right hemisphere.

Voxel-based morphometry analysis of the 3D T1-weighted images suggested that GM density in the left olfactory cortex, right orbital superior frontal gyrus, bilateral insula, left globus pallidus, and temporal lobe region (left superior temporal gyrus temporal pole, bilateral middle temporal gyrus temporal pole, and right middle temporal gyrus) of HA group was significantly lower than that HCs in the LA group (Table 3 and Figures 1–3).

**TABLE 3:** Comparison of average gray matter density in each brain area between the two groups.

ROIs name (AAL_Label)	Mean GM density (SD)		*t*-value	*p*-value
	HA	LA		
Olfactory_L (21)	0.514 (0.073)	0.535 (0.061)	−3.824	<0.001
Frontal_Med_Orb_R (26)	0.418 (0.069)	0.444 (0.047)	−3.779	<0.001
Insula_L (29)	0.450 (0.063)	0.475 (0.044)	−4.604	<0.001
Insula_R (30)	0.476 (0.068)	0.500 (0.043)	−4.004	<0.001
Cingulum_Ant_L (31)	0.436 (0.069)	0.479 (0.048)	−4.301	<0.001
Pallidum_L (75)	0.249 (0.030)	0.270 (0.032)	−4.038	<0.001
Temporal_Pole_Sup_L (83)	0.379 (0.054)	0.400 (0.046)	−3.887	<0.001
Temporal_Mid_L (85)	0.393 (0.048)	0.411 (0.042)	−4.178	<0.001
Temporal_Mid_R (86)	0.403 (0.056)	0.419 (0.037)	−3.766	<0.001
Temporal_Pole_Mid_L (87)	0.419 (0.056)	0.462 (0.049)	−5.203	<0.001
Temporal_Pole_Mid_R (88)	0.376 (0.046)	0.404 (0.044)	−4.184	<0.001

*Notes: VBM analysis: HA vs. LA group, P < 0.05, Bonferroni correction.*

**FIGURE 4:**
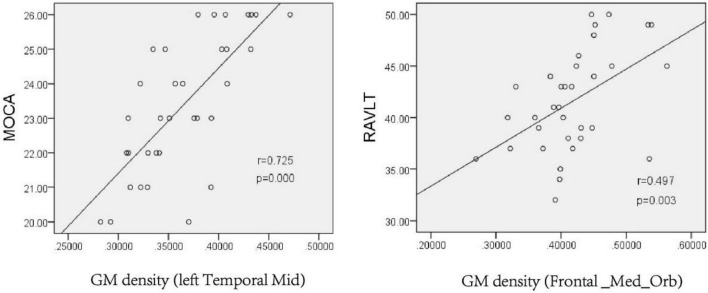
Pearson analysis of neuropsychological scale score and gray matter density in the left Temporal Mid and Frontal_Med_Orb after chronic hypoxia exposure at high altitude (*p* < 0.05). MoCA, montreal cognitive assessment scale; RAVLT, rey auditory verbal learning test.

Pearson correlation analysis was conducted on the GM density of relevant brain regions and the neuropsychological scale scores. The results showed that the GM density of the left middle temporal gyrus was positively correlated with the MoCA scale score after chronic hypoxia exposure at high altitude (*r* = 0.725, *P* < 0.001). There was also a positive correlation between the GM density of the right superior frontal gyrus and RAVLT score (*r* = 0.497, *P* = 0.003), suggesting that decreased GM density in related brain regions may be the structural basis of cognitive impairment (Figure 4).

#### Changes in the White Matter Fiber Connections

**FIGURE 5:**
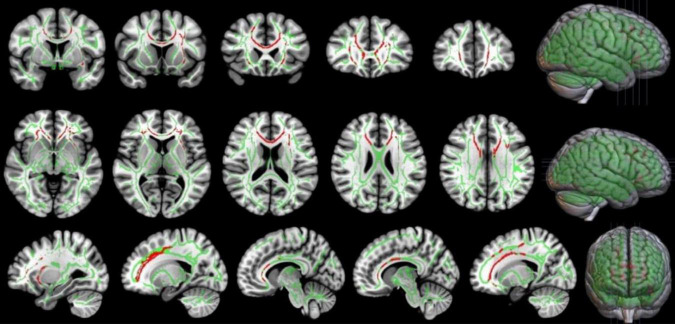
Comparison of FA in local brain regions of the HA and LA participants. Red represents clusters of brain regions with reduced FA values after chronic hypoxia exposure. Green represents the average FA bundle skeleton template. TBSS: HA<LA (*p* < 0.05, TFCE corrected). Threshold-Free Cluster Enhancement, TFCE, is a Multiple comparison correction method. By this method, the FA values of the voxels on the fiber bundle skeleton of the two groups were compared, and the regions with significant inter-group differences were highlighted.

In this study, TBSS was used to analyze the DTI data. Two-sample *t*-test results showed that, compared with the LA group, the HA group had lower FA in the corpus callosum, corpus callosum knee, bilateral corona radiata, and left internal capsule area (*p* < 0.05, TFCE corrected), as shown in Figure 5 and Table 4.

**TABLE 4:** MNI coordinates of the corpus callosum showing FA decreases due to chronic hypoxia.

Cluster name	Number of voxels	Peak FA	Peak MNI coordinate			Center of mass		
			*x*	*y*	*z*	*x*	*y*	*z*
Body of corpus callosum	7,406	0.993	−14	22	22	−4.49	19.1	15.8

*The numbers in the table show the number of corpus callosum voxels and the peak value of FA, while other values show the size of the corresponding coordinate axes.*

**FIGURE 6:**
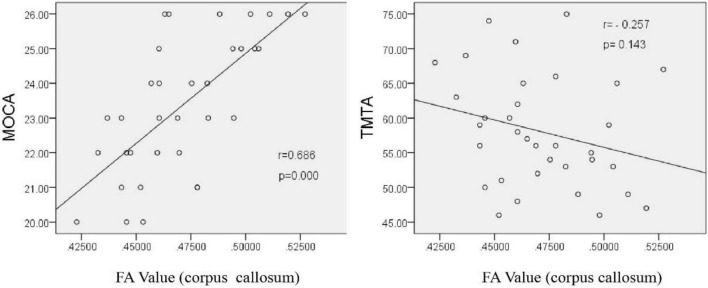
Correlation analysis between the corpus callosum FA value and neuropsychological scale scores after chronic hypoxia exposure in the HA group (*p* < 0.05).

Pearson correlation analysis showed that the FA value of the corpus callosum was positively correlated with MoCA score (*r* = 0.686, *P* < 0.001), but had no significant correlation with TMTA score (*r* = −0.257, *P* = 0.143). These results suggest that the incompleteness of WM fibers in the corpus callosum may be an important structural basis for the decline of overall cognitive function, but has no direct relationship with the decline of executive function (Figure 6).

### Functional Magnetic Resonance Imaging Comparison

**FIGURE 7:**
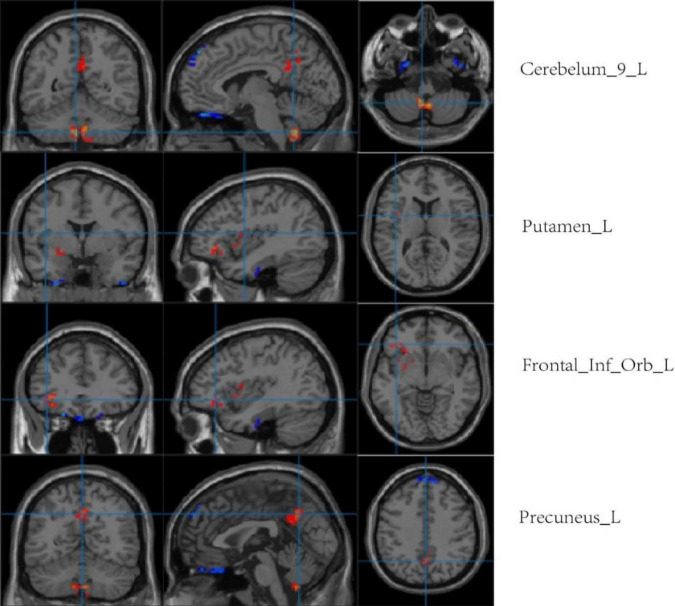
Regional comparison of ALFF values after chronic hypoxia exposure (*p* < 0.001, cluster size > 50). Red represents brain regions with reduced ALFF after chronic hypoxia exposure (HA<LA). Blue represents brain regions exposed to chronic hypoxia with elevated ALFF (HA>LA).

Compared with the LA group, the HA group had lower ALFF values in the left cerebellum, left putamen, left orbital inferior frontal gyrus, and left precuneus. There was no significant difference in ReHo values between the two groups (*p* < 0.001, cluster size > 50; Figure 7 and Table 5).

**TABLE 5:** MNI coordinates of brain regions showing significant changes in ALFF/ReHo values after chronic hypoxia exposure at high altitude.

Cluster name	Number of voxels	Peak intensity	Peak MNI coordinate		
			*x*	*Y*	*z*
Cerebelum_9_L	89	7.356	−6	−54	−48
Fusiform_L/Temporal_Inf_L	87	−5.675	−33	−9	−39
Temporal_Inf_R	56	−4.997	48	−18	−33
Frontal_Sup_Orb_L	96	−5.98	−3	48	−27
Putamen_L	64	4.657	−36	3	12
Frontal_Inf_Orb_L	55	4.643	−39	33	−6
Precuneus_L	90	4.911	0	−60	39
Frontal_Sup_Medial_L	51	−4.878	−9	51	48

*Notes: LA-HA (p < 0.001, cluster size > 50). For details see section “rs-fMRI.”*

**FIGURE 8:**
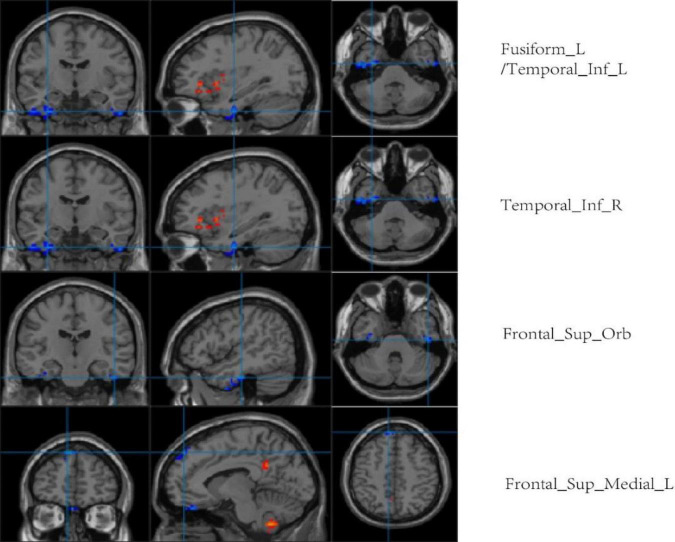
Regional comparison of ALFF values after chronic hypoxia exposure (*p* < 0.001, cluster size > 50). Red represents brain regions with reduced ALFF after chronic hypoxia exposure (HA<LA). Blue represents brain regions of individuals exposed to chronic hypoxia with elevated ALFF (HA>LA).

Compared with the LA group, ALFF values of the left fusiform gyrus, bilateral inferior temporal gyrus, left orbital superior frontal gyrus, and medial superior frontal gyrus were higher in the HA group. There was no significant difference in ReHo values between the two groups (*p* < 0.001, cluster size > 50; Figure 8 and Table 5).

**FIGURE 9:**
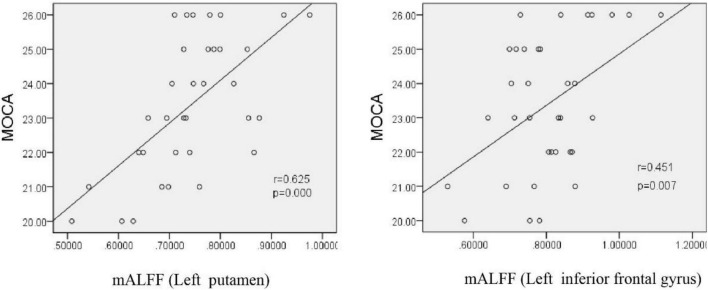
Correlation between the mALFF value and MoCa score in HA participants. MoCA, montreal cognitive assessment scale; mALFF, amplitude of low frequency fluctuations.

We normalized the ALFF value to obtain mALFF value, and then conducted correlation analysis. Pearson correlation analysis showed that the mALFF values of the left putamen and left inferior frontal gyrus were positively correlated with MoCA scale scores after exposure to chronic hypoxia at high altitude (*r* = 0.625, *P* = 0.000; *r* = 0.451, *P* = 0.007), suggesting that decreased neuronal activity in the above-mentioned brain functional areas may be the brain functional basis of cognitive decline (Figure 9).

## Discussion

At present, there are relatively few studies on the impact of plateau environment on the cognitive function of long-term residents. Furthermore, the findings of existing studies are susceptible to the impact of residence time, altitude, ethnic composition, and other factors, presenting inconsistent results (Hill et al., 2014; Das et al., 2018).

In the present study, it was found that the cognitive functions of people living at high altitude were impaired to varying degrees and in a wide range of domains. Impaired cognitive domains included memory function, executive function, visuospatial ability, attention, and language. Memory function is the main aspect of cognitive impairment induced by hypoxia. Both acute and chronic hypoxia can lead to memory impairment, including short-term and long-term impairment. However, unlike acute exposure, which mainly damages memory encoding function (Davis et al., 2015), chronic hypoxic exposure mainly damages memory storage and retrieval functions (Hornbein, 1992). For the RAVLT memory test in our study, both the immediate memory score and delayed recall score in the HA group were significantly reduced, but the delayed recall score was lower. These results indicate that chronic hypoxic exposure resulted in more significant damage to long-term memory, which is consistent with other research findings (Zhang et al., 2013). The decrease in memory function may be related to damage of the cerebral cortex and hippocampus.

Executive function is a complex domain involving a variety of higher brain functions such as planning, working memory, thinking flexibility, and psychomotor ability (Chen et al., 2021). Previous studies have shown that acute or chronic high-altitude exposure may lead to impaired executive function (Wei et al., 2021), and decreased executive function may be another important manifestation of cognitive impairment induced by chronic high-altitude hypoxic environment exposure. The results of the TMT and clock drawing test of MoCA showed that the HA group was significantly worse than the LA group in terms of connection speed and accuracy, as well as clock drawing accuracy, indicating that executive function was impaired to varying degrees. Currently, the neurobiological mechanism of executive function impairment induced by chronic hypoxia has not been elucidated; the neuroimaging evaluation conducted in this study may reveal the possible mechanism to some extent, as discussed in the following section.

Attention and visuospatial ability are also important aspects of cognitive evaluation. Decreased attention (sustained attention, selective attention, distributive attention, and processing speed) and visuospatial ability (visual sense and orientation) are two of the manifestations of cognitive impairment induced by a high-altitude hypoxic environment. Previous studies of people living in a high altitude and low oxygen environment (altitude of 3,700 m) in the Andes mountains for a long time found that attention and quick reaction ability was significantly decreased compared with people living at low altitude (Das et al., 2018). In the present study, the TMT A and B test results suggested that the time to complete the test in the HA group was longer than that in the LA group, while the DST results showed that the accurate numbers in the HA group were lower than those in the LA group. These results suggest that attention and visual spatial ability are influenced by a long-term low oxygen environment, as the HA group showed significantly worse test performance. The SDMT is mainly used to evaluate attention. However, we did not find any difference in SDMT scores between the two groups. This may be related to the low sensitivity of this attention test in the high-altitude environment, which suggests the need for more objective evaluation scales and cognitive testing tools at altitude. Further research is needed to clarify what neuropsychological evaluation tool is more sensitive to test changes in attention induced by a chronic hypoxic environment at a high altitude.

In this study, VFT was used to test the effect of chronic hypoxic exposure on language fluency. It was found that the VFT score of people living at a high altitude was significantly lower than that of the LA group, indicating a disorder in word fluency. Cognitive testing of Ecuadorian populations living at high altitudes found that semantic fluency was significantly impaired, which is consistent with the results of this study (Davis et al., 2015).

The test results of each scale were highly consistent with those of each item in the MoCA scale. This finding suggests that the impact of a chronic high altitude hypoxic environment on cognitive function is multi-faceted and involves domains of cognitive function, which is consistent with prior repots (Shanjun et al., 2020). According to the MoCA results, the incidence of cognitive dysfunction in the high-altitude population exposed to chronic hypoxia was 69.39%, while that in the low-altitude population was 37.21%, which is a statistically significant difference (*χ^2^* = 9.558, *p* = 0.002). Thus, higher altitude correlated with higher incidence of MCI.

It is worth noting the significant difference in ethnic composition between the two groups in this study due to differences in ethnic settlement in various areas of Qinghai. In the HA group, 59.18% of participants were Tibetan, while only 13.95% of participants in the LA group were Tibetan. Thus, whether ethnicity is a factor in cognitive decline should be clarified. Through a literature review, we found that an epidemiological survey of AD was carried out among the Tibetan population over 60 years old (*n* = 4,060 people) in Qinghai. The study found that the prevalence of AD in the Tibetan population was significantly lower than the national average (Huang et al., 2016). In the present study, although most of the Tibetan participants in the HA group were middle-aged or young, they still had significant cognitive impairment. Therefore, it is thought that cognitive decline in the HA group was induced by long-term exposure to a hypoxic environment at a high altitude, and there was no interference of the ethnic factor.

### Neuroimaging Measures

Whether the cognitive impairment induced by chronic hypoxic exposure at high altitude is the result of brain structure and function changes is a hot topic in brain science. In this study, we first applied VBM technology to analyze GM density changes between HA population and LA population. The results showed that the GM density in most temporal lobe regions, including the left superior temporal pole, bilateral middle temporal pole, and right middle temporal gyrus, was significantly reduced in HA population with chronic exposure to a high-altitude environment. GM density decreased in the left olfactory cortex, right medial orbital superior frontal gyrus, bilateral insula, and left globus pallidus. Decreased GM density is a quantitative indicator of brain atrophy, indicating loss of neurons and thinning of the cerebral cortex. The temporal lobe is the functional brain region of working memory, language, emotion, and other cognitive domains (Amoruso et al., 2018). Thus, temporal lobe damage is closely associated with cognitive impairment. The superior frontal gyrus is involved in a variety of brain functions including movement, emotion, memory, etc. (Hiser and Koenigs, 2018; Briggs et al., 2020), especially working memory. Damage to the superior frontal gyrus may lead to short-term memory storage and retrieval disorders. In this study, GM density was decreased in most areas of the temporal lobe, especially the bilateral middle temporal gyrus, and was positively correlated with MOCA score reflecting overall cognitive function. In addition, decreased GM density in the right superior frontal gyrus was positively correlated with RAVLT score, which mainly reflects working memory. This finding suggests an internal correlation between imaging results and neuropsychological measures. In the HA population, the GM density of the left olfactory cortex, bilateral insula, left globus pallidus, and other brain areas also decreased significantly. We believe that the extensive decrease in GM density may be the main structural basis for cognitive impairment induced by high altitude exposure. Although there are no relevant reports on the mechanism of GM density reduction caused by chronic hypoxia at high altitude, some scholars believe that GM density reduction may be related to brain tissue metabolites after hypoxia (Zatorre et al., 2012). In previous animal experiments (Ji et al., 2021a,b), we found that chronic hypoxic exposure in rats led to increases in lipid peroxides, free radicals, and oxidized glutathione. Whether similar changes occur in human brain tissue following chronic hypoxic exposure will likely become a future research direction.

In the present study, the TBSS method was used to analyze DTI imaging data of patients with cognitive impairment induced by chronic hypoxic exposure (HA group). HA population had lower FA in the corpus callosum, corpus callosum knee, bilateral radiographic corona, and left internal capsule region. However, previous studies comparing DTI data of plateau immigrants and plains people found that the FA values of bilateral radiative corolla, bilateral internal sacs, and bilateral external sacs were higher in plateau immigrants, which was believed to be caused by the thickening of the phospholipid myelin sheath of nerve fibers after exposure to a low oxygen environment (Yan et al., 2010). The results of the current study showed decreased FA values in several brain regions after chronic hypoxic exposure, which may be related to nerve fiber damage, edema, and morphological and structural changes in axons. The corpus callosum, as an important fibrous structure connecting the two cerebral hemispheres, plays an important role in coordinating bilateral brain functions. The bilateral corolla radiata and inner capsule are key relay stations for nerve information transmission, and their upper and lower nerve fibers mediate the transmission of nerve impulses up and down. Therefore, it is speculated that damage of nerve fibers revealed by DTI in the above brain regions may affect the conduction of nerve impulses to a certain extent, thus indirectly affecting cognitive function. Notably, there was a significant positive correlation (*r* = 0.686, *p* < 0.001) between the FA value of the corpus callosum and MOCA scale score in the present study, indicating that damage of nerve fibers in the corpus callosum under hypoxic conditions affected cognitive function. Therefore, we believe that local nerve fiber damage, as reflected by decreased FA value, may be an important structural basis for cognitive impairment induced by chronic hypoxic exposure at high altitude. In contrast, no significant correlation was found between the FA value of the corpus callosum and TMTA score, which mainly reflects attention (*r* = −0.257, *P* = 0.143). This result may reflect that the functional basis of the corpus callosum does not involve attention, but is associated with overall cognitive impairment. Owing to various interfering factors, more DTI parameters were not statistically analyzed in this study.

rs-fMRI has been widely used in previous cognitive studies. A study that compared college students living in a plateau area with those living in a plain area found an enhanced blood-oxygen-level-dependent (BOLD) signal in multiple brain regions of people living in a plateau area (Yan et al., 2011b). As expected, BOLD signal enhancement was consistent with ReHo and ALFF values. ReHo and ALFF are both important features for describing rs-fMRI data. It is believed that ReHo mainly reflects the similarity or consistency of the time series in a functional region, i.e., the consistency of activity pace describing adjacent voxel regions. ALFF mainly reflects the degree of spontaneous neural activity in a period of time, i.e., it describes the activity intensity of single voxel region. An increase in ReHo value indicates an increase in neuronal activity that is more uniform and consistent over time, while a decrease in ReHo value indicates disorder of neuronal activity in local brain functional area and disorder in the time series, which may indicate serious brain dysfunction (Chen et al., 2019). In this study, the ReHo values between the HA and LA groups showed no obvious difference. Consider related to HA group is given priority to with HA population, high altitude in spite of MCI in population with HA, neuron activity time failed to maintain uniform consistency, but has not yet appeared severe brain dysfunction, so the ReHo values are almost consistent with the LA group. This is not consistent with Other related studies. It is not clear whether this difference is related to altitude. An increase in ALFF value indicates the enhancement of spontaneous neuronal activity in a region, while a decrease in ALFF value indicates weakening of spontaneous neuronal activity in a region. In the present study, compared with the LA group, the ALFF values of the left cerebellum, left putamen, left orbital inferior frontal gyrus, and left precuneus were lower in the HA group. Furthermore, the ALFF values of the left putamen and left inferior frontal gyrus were positively correlated with MoCA scale scores (*r* = 0.625, *P* = 0.000; *r* = 0.451, *P* = 0.007, respectively). It is worth noting that, in addition to structurally carrying out the important relay functions of upstream and downstream fibers, the putamen is likely to be involved in complex cognitive processes such as learning and working memory (Shu et al., 2009; Baier et al., 2010). Therefore, the functional abnormalities reflected by the decreased ALFF value in the putamen region in this study may partially explain the impairment in cognitive function caused by chronic hypoxia. The positive correlation between left putamen functional abnormalities and overall cognitive function support this explanation. The extensive decreases in ALFF values indicate that the spontaneous activity of neurons in multiple brain regions was decreased by long-term hypoxic exposure, which may be the functional basis of the overall cognitive decline in people living at high altitude. Interestingly, this study also found that the ALFF values of other brain regions, such as the left fusiform gyrus, bilateral inferior temporal gyrus, left orbital superior frontal gyrus, and medial superior frontal gyrus, were relatively higher in the HA group compared to the LA group, indicating enhanced spontaneous brain activity in these regions. Each voxel in the brain represents an ALFF value, and each ALFF value represents the activity intensity of the voxel in the brain. Therefore, the activity intensity of each voxel is different. On this basis, we speculate that the inconsistent intensity of neuronal activity in different brain regions after chronic hypoxic exposure may be related to the richness of local blood supply and different tolerances to hypoxia (Wise et al., 2010; Perosa et al., 2020). As the neuronal structure, receptors, and neurotransmitters of GM in different brain regions are different, the tolerance and response of different brain regions to factors such as hypoxia may also be different (Chang et al., 2006). Further research is warranted to shed light on this phenomenon.

## Data Availability Statement

The raw data supporting the conclusions of this article will be made available by the authors, without undue reservation.

## Ethics Statement

The studies involving human participants were reviewed and approved by Medical Ethics Committee of Qinghai Provincial People’s Hospital. The patients/participants provided their written informed consent to participate in this study. Written informed consent was obtained from the individual(s) for the publication of any potentially identifiable images or data included in this article.

## Author Contributions

YZ: methodology, validation, investigation, neuropsychological testing, statistical analysis, and writing – original draft. WJ: conceptualization, supervision, and writing – review and editing. WZ: methodology, investigation, and data curation. JL: investigation, imaging evaluation, data processing, and data curation. All authors contributed to the article and approved the submitted version.

## Conflict of Interest

The authors declare that the research was conducted in the absence of any commercial or financial relationships that could be construed as a potential conflict of interest.

## Publisher’s Note

All claims expressed in this article are solely those of the authors and do not necessarily represent those of their affiliated organizations, or those of the publisher, the editors and the reviewers. Any product that may be evaluated in this article, or claim that may be made by its manufacturer, is not guaranteed or endorsed by the publisher.

## Footnotes

1
https://www.fil.ion.ucl.ac.uk/

2
http://dbm.neuro.uni-jena.de/cat12/

3
https://fsl.fmrib.ox.ac.uk/fsl/fslwiki/

4
http://rfmri.org/DPABI
